# Relationships between Micro-Vascular and Iodine-Staining Patterns in the Vicinity of the Tumor Front of Superficial Esophageal Squamous Carcinoma

**DOI:** 10.1371/journal.pone.0126533

**Published:** 2015-08-24

**Authors:** Shunsuke Ohta, Kenro Kawada, Jirawat Swangsri, Naoto Fujiwara, Katsumasa Saito, Hisashi Fujiwara, Tairo Ryotokuji, Takuya Okada, Yutaka Miyawaki, Yutaka Tohkairin, Yasuaki Nakajima, Youichi Kumagai, Kagami Nagai, Takashi Ito, Yoshinobu Eishi, Tatsuyuki Kawano

**Affiliations:** 1 Department of Esophageal and General Surgery Tokyo Medical and Dental University Yushima 1-5-45, Bunkyo-ku, Tokyo 113-8519, Japan; 2 Department of Human Pathology Tokyo Medical and Dental University Yushima 1-5-45, Bunkyo-ku, Tokyo, 113-8519, Japan; West German Cancer Center, GERMANY

## Abstract

**Objective:**

The aim of the present study was to clarify differences between micro-vascular and iodine-staining patterns in the vicinity of the tumor fronts of superficial esophageal squamous cell carcinomas (ESCCs).

**Methods:**

Ten consecutive patients with ESCCs who were treated by endoscopic submucosal dissection (ESD) were enrolled. At the edge of the iodine-unstained area, we observed 183 sites in total using image-enhanced magnifying endoscopy. We classified the micro-vascular and iodine-staining patterns into three types: Type A, in which the line of vascular change matched the border of the iodine-unstained area; Type B, in which the border of the iodine-unstained area extended beyond the line of vascular change; Type C, in which the line of vascular change extended beyond the border of the iodine-unstained area. Then, by examining histopathological sections, we compared the diameter of intra-papillary capillary loops (IPCLs) in cancerous areas and normal squamous epithelium.

**Results:**

We investigated 160 sites that the adequate quality of pictures were obtained. There was no case in which the line of vascular change completely matched the whole circumference of the border of an iodine-unstained area. Among the 160 sites, type A was recognized at 76 sites (47.5%), type B at 79 sites (49.4%), and type C at 5 sites (3.1%). Histological examination showed that the mean diameter of the IPCLs in normal squamous epithelium was 16.2±3.7μm, whereas that of IPCLs in cancerous lesions was 21.0±4.4μm.

**Conclusions:**

The development of iodine-unstained areas tends to precede any changes in the vascularity of the esophageal surface epithelium.

## Introduction

For successful cure of esophageal squamous carcinoma (ESCC), detection at an early stage is essential. However, it has not been easy to diagnose early-stage ESCC using conventional endoscopy [1]. Chromoendoscopy using iodine-staining is a very sensitive screening technique, and has markedly improved the rate of early detection of ESCC [2],[3]. However, iodine-staining has certain drawbacks: it prolongs the time required for screening, some patients may have iodine allergy, and it can elicit certain side effects such as chest discomfort or coughing[2],[4].

Recently, several studies have demonstrated the usefulness of image-enhanced magnifying endoscopy for detection of early-stage ESCC, determination of the depth of cancer invasion, and assessment of the degree of cancer spread [5],[6].

Magnifying endoscopic observation of the normal esophageal mucosa and ESCC was first reported by Inoue et al. [5],[7], who succeeded in recognizing looped capillary vessels inside the epithelial papillae (intra-papillary capillary loops: IPCL). The IPCL or IPCL-like vessels inside cancerous lesions were demonstrated to show abnormal changes such as dilation, weaving, changes in caliber, and variety of shape. Also, the morphology of the surface vasculature of superficial ESCC exhibited characteristic changes according to the depth of tumor invasion.

Some groups have reported that image-enhanced magnifying endoscopy is able to detect the border of cancer clearly by visualizing the so-called “brownish area” [8], and have considered that this is advantageous in comparison with iodine-staining, and its various unfavorable aspects [2],[4],[9]. However, the relationship between changes in micro-vascular morphology and the iodine-unstained area of mucosal cancer has not been investigated.

In the present study, we compared morphological changes in IPCLs and the iodine-unstained area in the vicinity of mucosal ESCC. Also, using histopathological sections, we compared the diameter of IPCLs in cancerous areas and normal squamous epithelium.

## Materials and Methods

Ten consecutive patients with ESCC who were treated by endoscopic submucosal dissection (ESD) between December 2012 and March 2013 at Tokyo Medical and Dental University were enrolled in this study. To assess the margins of iodine-unstained areas, we observed the entire circumference of the resected specimens at regular intervals using image-enhanced magnifying endoscopy. We observed total 183 points in all (an average of 20 points in each case). At 12 points it was not possible to observe the microvasculature in the cancerous or non-cancerous area because of scant pooled blood in the resected specimen, whereas at 11 points this was not possible because of the poor condition of the chosen areas due to cornification and inflammation. Thus, the number of areas evaluated was 160 (87.4%).

For this research, we employed a magnifying endoscope EG-L590ZW (FUJI　FILM Co., Tokyo) with a LASEREO system (VP4450 HD, FUJI FILM) with a light source (LL4450; FUJI FILM).

We observed the resected specimens under high magnification (x100) in blue laser imaging (BLI) mode to evaluate the micro-vascular morphology, followed by iodine-staining just after the ESD procedure. For magnifying observation using iodine-staining, we employed sodium thiosulfate (Detoxol) repeatedly to counteract the influence of the iodine. We focused on the intra-papillary capillary loops (IPCLs) and observed the morphological changes in these capillaries at the border of the tumor with the adjacent normal squamous epithelium. First, we observed IPCLs from the adjacent normal squamous epithelium towards the border of the iodine-unstained area. We defined the border at which the IPCLs showed a recognizable cancerous change as the “line of vascular change”. Changes in vascular structure were considered in terms of both morphology (dilation and elongation) and the density of IPCLs (S1 Fig).

The relationship between the iodine-unstained area and the morphology of the microvasculature was classified into three types as follows:

Type A: The line of vascular change matched the border of the iodine-unstained area (S3 Fig). Thus, the border of the iodine-unstained area completely matched that of the morphological change in the microvasculature.
Type B: The border of the iodine-unstained area extended widely beyond the line of vascular change (S4 Fig).
Type C: The line of vascular change extended widely beyond the border of the iodine-unstained area (S5 Fig).

Four endoscopists (S.O, K.K, Y.K, and T.K.) discussed and judged the findings recorded on video together and determined the relationship between the change in the vascular line and the border of the iodine-unstained area on the basis of the above classification.

From histological sections, we measured the diameter of microvessels at the border between the normal squamous epithelium and ESCC for each of types A, B, and C, taking into consideration the plane of section in resected specimens and the pictures obtained by magnifying endoscopy. We measured the diameter of normal and cancerous vessels inside the mucosal papillae (IPCLs) as close as possible to the border between the normal and cancerous areas. The average diameters of normal and cancerous IPCLs in each of types A, B, and C were calculated and compared.

Variables were expressed as mean±standard deviation (SD). Differences between groups were analyzed by *t* test or paired *t* test. Differences at P <0.05 were considered significant.

This study was approved by the ethics committee of Tokyo Medical and Dental University (No. 1375).

## Results

There were no cases in which all of the observed points were type A, i.e., in none of the cases did the vasculature line completely match the border of the iodine-unstained area around the entire circumference.

Among the 160 points evaluated, type A was recognized at 76 points (47.5%), type B at 79 points (49.4%), and type C at 5 points (3.1%). The median distance from the border of the iodine-unstained area to the line of vascular change was 0.89 mm (range: 0.38–1.96 mm) for type B (S6 Fig), and 1.16 mm (range: 0.8–2.52 mm) for type C (S7 Fig). At 68 points (42.5%) representing all types, alteration of the vascular morphology was the main factor determining the line of vascular change, whereas an increase of vascular density was the main factor at 39 points (24.4%) and changes in both vascular morphology and vascular density were responsible at 53 points (33.1%) (Table 1).

**Table 1:** Incidence of features observed in each type of vascular relationship.

	Alternation of vascular morphology cases (%)	Increase in vascular density cases (%)	Both factors cases (%)
Type A	37 (48.7)	13 (17.1)	26 (34.2)
Type B	30 (38.0)	23 (29.1)	26 (32.9)
Type C	1 (20.0)	3 (60.0)	1 (20.0)
	68 (42.5)	39 (24.4)	53 (33.1)

On the basis of histological examination, 51 sections in 10 cases were classified as type A, 36 sections in 10 cases as type B, and 2 sections in 2 cases as type C. The mean diameter of IPCLs in normal squamous epithelium was 16.2±3.7 μm (mean±SD, n = 288), whereas that in cancerous areas was 21.0±4.4 μm (n = 288), and the difference was significant (P<0.001) (S8 Fig). When this analysis was conducted on the basis of micro-vascular and iodine-staining patterns, in type A the mean diameter of IPCLs in normal squamous epithelium was 16.0±4.0 μm, whereas that of IPCLs in cancerous areas was 22.2±4.2 μm, the difference being significant (P<0.001) (S9 Fig). In type B, the corresponding figures were 16.5±1.7 μm and 16.5±1.4 μm, respectively, and difference was not significant (P = 0.35) (S10 Fig).

The two sections classed as type C were confirmed to be basal layer-type ESCC histologically (S11 Fig). The mean diameter of IPCLs was 18.5±1.29 μm in the normal squamous epithelium and 21.3±0.95 μm in the cancerous area. However, as only two sections were available for type C, statistical analysis was not possible.

## Discussion

The normal esophageal epithelium possesses a number of glycogen granules located mainly in the prickle cell layer above the basal cell layer. It is well known that these glycogen granules are darkly stained by iodine. However, in malignant or dysplasia-related epithelium, these granules are reduced or absent, thus allowing such lesions to be detected as unstained areas using iodine chromo-endoscopy [10].

Generally, in a clinical setting, the iodine-unstained area is considered to correspond to the area that is histologically cancerous. However, precise comparison between the two areas is quite difficult because of problems with histopathological definition, which arise due to differences in histological interpretation between Western and Eastern pathologists [11]. For this reason, our present study was focused purely on clinical observation, and no attempt was made to obtain precise pathological diagnoses.

Recently, image-enhanced magnifying endoscopy using narrow-band imaging (NBI) has made it possible to detect early-stage ESCC [5],[6],[8]. This technique has also been adopted for determining the depth of ESCC invasion based on morphological alterations resulting from vertical tumor extension [12],[13], and some investigators have shown that it is also useful for determining the margin of ESCC in comparison with mucosal iodine-staining observed using conventional white light endoscopy [14].

However, it has not been investigated whether the areas diagnosed as cancerous by image-enhanced magnifying endoscopy on the basis of a change in the microvasculature do, in fact, match the iodine-unstained areas of the mucosa, hitherto considered to have been the gold standard for delineating the area of ESCC.

It is considerably disadvantageous that the mucosal microvasculature cannot be well visualized in surgically resected esophageal specimens because of insufficient pooling of intra-micro-vascular blood. In this context, Kumagai et al. have introduced the use of MICROFIL (Flow Tech, Inc., Tokyo), which is injected into the microvessels of the resected esophagus to allow observation using stereoscopic microscopy [15],[16]. This technique has made it possible to compare the microvasculature of the surface epithelium with histologic findings. However, the technique for injecting MICROFIL into small esophageal vessels is difficult and time-consuming.

In the present study, we applied the LASERREO system to specimens that had been resected by ESD, and succeeded in observing the mucosal microvasculature, similarly to endoscopic observation in vivo.

The LASERREO system (Fuji Film Co., Tokyo) is a novel endoscopic system that uses a laser light source. It employs lasers of two wavelengths, one for white light observation (wavelength 450±10 nm), and the other for blue light imaging (BLI) (wavelength: 410±10 nm). The BLI facilitates clear enhancement of the surface microvasculature using narrow-band laser light.

In the ESD procedure, we cut and coagulated the microvasculature present in the lamina propria mucosae or submucosa at the resection margin, and also carefully cut the perforated vessels from the muscle layer. This caused blood to remain in the micro-vessels, thus allowing us to perform endoscopic observation similar to that in a clinical setting. In this way, we were able to perform detailed evaluation of the correlation between morphological changes in the microvasculature and areas unstained by iodine in superficial esophageal carcinomas.

For magnifying endoscopic observation of the esophagus, recognition of IPCLs is essential. IPCLs present in the normal esophageal mucosa are terminal capillaries that arise from the subepithelial capillary network in the lamina propria mucosae and extend toward the epithelial papillae [5],[7],[13],[15],[17]. In the normal epithelium, IPCLs are arranged regularly at intervals of about 100 μm, which corresponds to the maximum distance across which oxygen can diffuse from a vessel [16],[17].

Our histological examinations showed that the diameter of IPCLs was 16.2±3.7 μm in the normal squamous epithelium and 21.0±4.4 μm in cancerous areas, thus being significantly thicker in the latter. This finding supports the observation that IPCLs in cancerous lesions appear to be dilated, and this situation may lead to changes in the morphology of IPCLs such as “weaving” or variations of shape [5],[13],[15]. Our histological observations also revealed that in the type A mucosal pattern, where the iodine-unstained area matched the cancerous change in the microvasculature, the IPCLs in the cancerous area were significantly thicker than those in the adjacent normal squamous epithelium. On the other hand, in the type B pattern, where the iodine-unstained area extended beyond the area of cancerous change in the microvasculature, the diameter of the IPCLs showed no significant difference between the two areas. These findings matched our BLI observations of resected specimens. Our investigation also revealed that the type B pattern was most frequent, followed by type A. Considering these findings as a whole, it appears that histological changes tend to precede any changes in the microvasculature. In other words, changes in vascular morphology occur secondarily in response to alterations in oxygen or nutritional demand resulting from a cancerous change in histology.

On the other hand, 5 (3.1%) of the 160 points we observed were determined as type C, in which the area showing a cancerous change in the microvasculature extended beyond the iodine-unstained area. These cases were confirmed to be basal layer-type ESCC histologically, and we also confirmed that the IPCLs in the cancerous area tended to be dilated in comparison with those in the normal squamous epithelium. In basal layer-type ESCC, atypical cells are located at, or adjacent to, the basal layer of the squamous epithelium. This makes it difficult to accurately recognize the histological border, as the surface squamous cells above the basal layer cancer are stained with iodine [18]. In type C, the distance from the line defined by the microvasculature to the border of the iodine-unstained area was up to 2.52 mm. As type C accounted for 2 of 10 cases, the incidence rate was considered to be high.

We also defined the cancerous change in the microvasculature in terms of the density of IPCLs in tandem with vascular morphology. In 60% of all the areas we observed, the IPCL density was increased. Among these areas, approximately 30% were considered to show a “cancerous change” in the microvasculature, due mainly to an increase of IPCL density without any morphological change. In this connection, it has been reported previously that CD105-positive IPCLs (neovasculature) are already present in low-grade dysplasia, and that microvessel density in cancerous lesions is significantly increased in comparison with normal squamous epithelium [19],[20],[21]. CD 105 is the most reliable marker of epithelial cell proliferation, and it is known to be expressed strongly in tumor vessels [19]. Such observations accord with the increased IPCL density we found in the present study.

Our present findings suggest that, at the borderline area between cancer and the adjacent normal squamous epithelium, histological changes precede or matched the morphological changes in the superficial microvasculature in the vast majority of cases. In some areas, however, the morphological change in the microvasculature extends beyond the iodine-unstained area. We should recognize certain risk for existence of the basal layer-type squamous cell carcinoma. We believe that our present data will facilitate a better understanding of the correlation between histological changes and angiogenesis in the early phase of cancer progression.

## Supporting Information

S1 Fig We defined the line where a cancerous change was evident in IPCLs as the “line of vascular change”. (TIFF)

S2 Fig When we observed IPCLs from the right upper side (normal squamous epithelium) towards the left side, we first recognized a change of density (a-1), and then we recognized the vascular morphology (a-2). (TIFF)

S3 Fig Type A: The line of vascular change matched the border of the iodine-unstained area. (TIFF)

S4 Fig Type B: The border of the iodine-unstained area extended widely beyond the line of vascular change. (line a: vascular line, line b: border of the iodine-unstained area)
(TIFF)

S5 Fig Type C: The line of vascular changed extended widely beyond the border of the iodine-unstained area. (TIFF)

S6 Fig a: Line of vascular change, b: Border of the iodine-unstained area, d: Distance from a to b. In type B, median d was 0.89 mm (range: 0.38–1.96 mm).
(TIFF)

S7 Fig In type C, median d was 1.16 mm (range: 0.8–2.52 mm). (TIFF)

S8 Fig Overall diameter of IPCL in normal squamous epithelium and ESCC. Normal IPCL: 16.2±3.7 μm (mean±SD). Cancerous IPCL: 21.9±4.4 μm (mean±SD) P<0.001, IPCL: Intra-papillary capillary loop.
(TIFF)

S9 Fig Diameter of normal IPCL and cancerous IPCL (Type A). Normal IPCL: 16.0±4.0 μm (mean±SD), Cancerous IPCL: 22.2±4.2 μm (mean±SD) P<0.001, IPCL: Intra-papillary capillary loop.
(TIFF)

S10 Fig Diameter of normal IPCL and cancerous IPCL (Type B). Normal IPCL: 16.5±1.7 μm (mean±SD) Cancerous IPCL: 16.5±1.4 μm (mean±SD) P = 0.35, IPCL: Intra-papillary capillary loop.
(TIFF)

S11 Fig All of the type C cases were confirmed to be basal layer-type ESCC histologically in the area of cancerous microvasculature stained with iodine. (TIFF)
