# Supplementary figures and images for: Relationships between Micro-Vascular and Iodine-Staining Patterns in the Vicinity of the Tumor Front of Superficial Esophageal Squamous Carcinoma
Source: PLoS One. 2015 Aug 24;10(8):e0126533. doi: 10.1371/journal.pone.0126533 (PMC4547752; doi:10.1371/journal.pone.0126533)

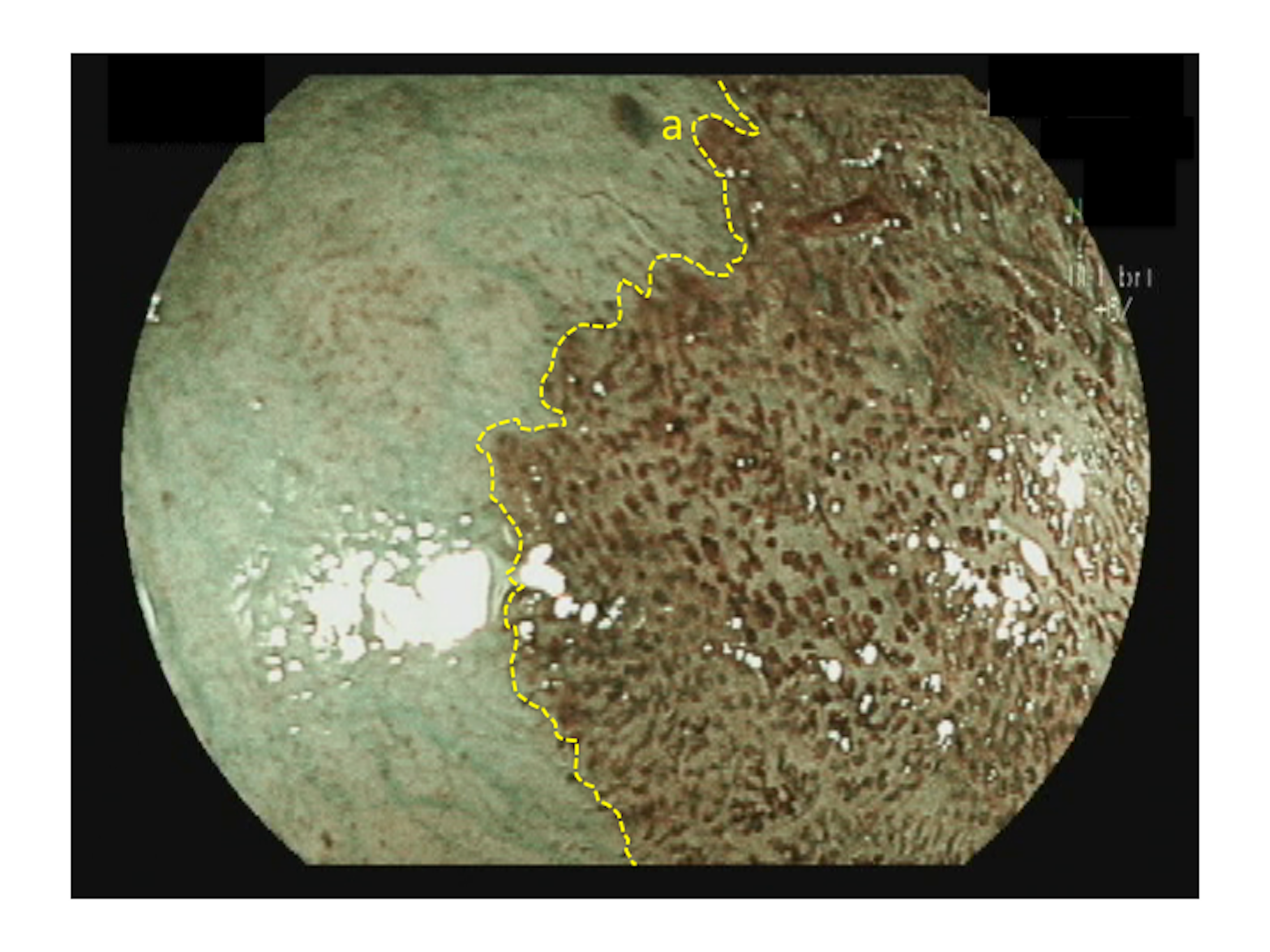

Supplement: S1 Fig — (TIFF) [file pone.0126533.s001.tiff]

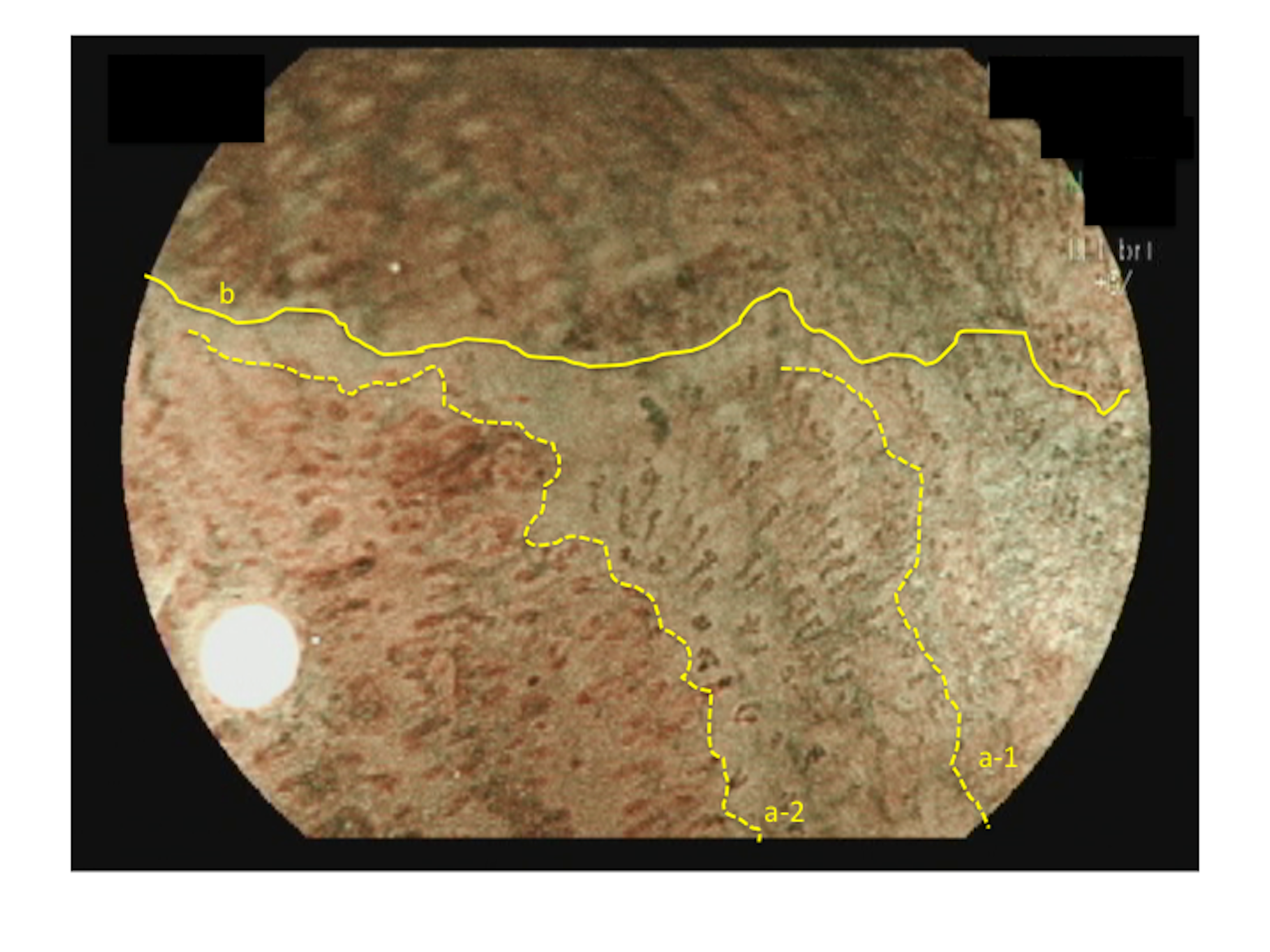

Supplement: S2 Fig — (TIFF) [file pone.0126533.s002.tiff]

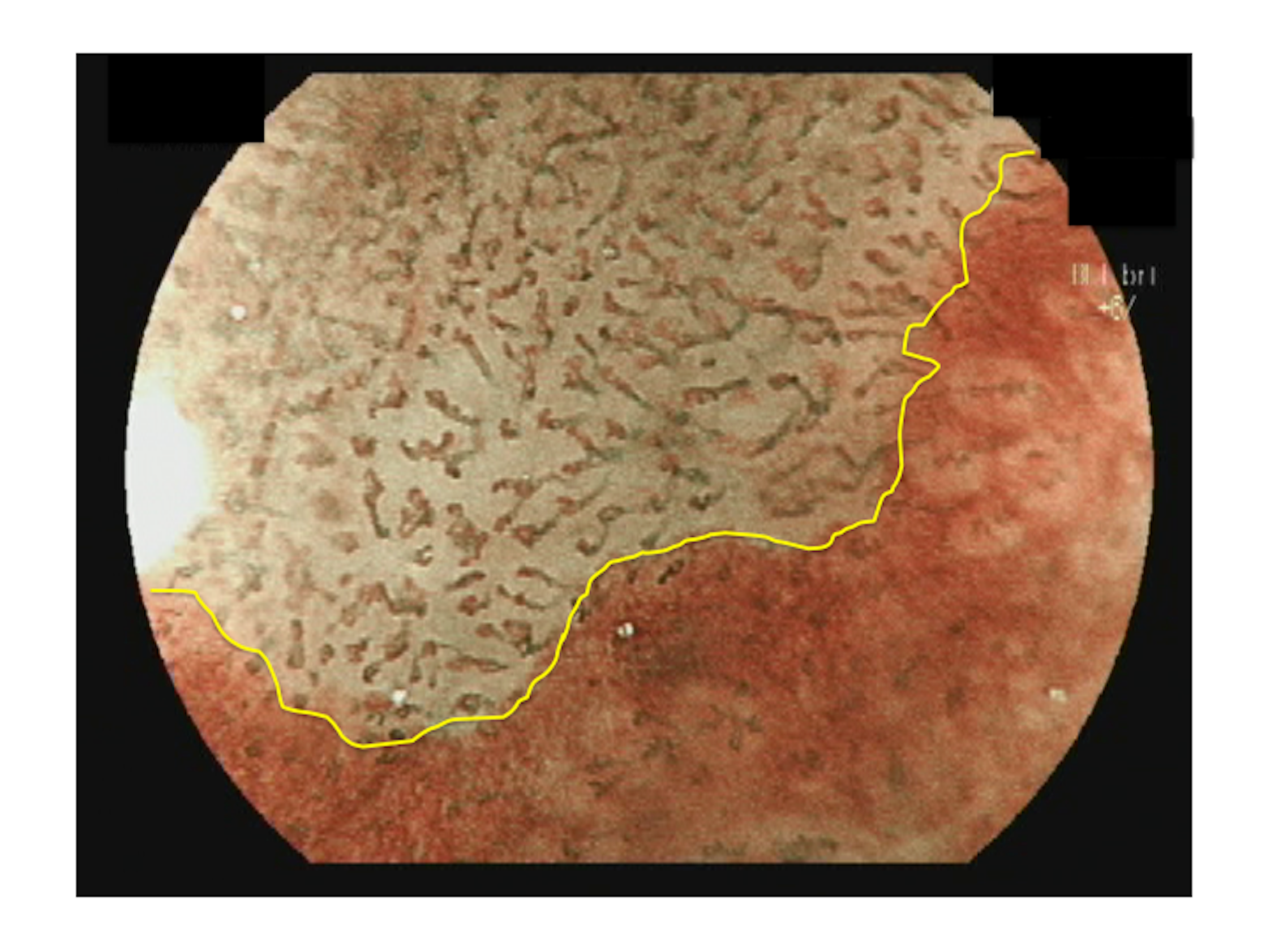

Supplement: S3 Fig — (TIFF) [file pone.0126533.s003.tiff]

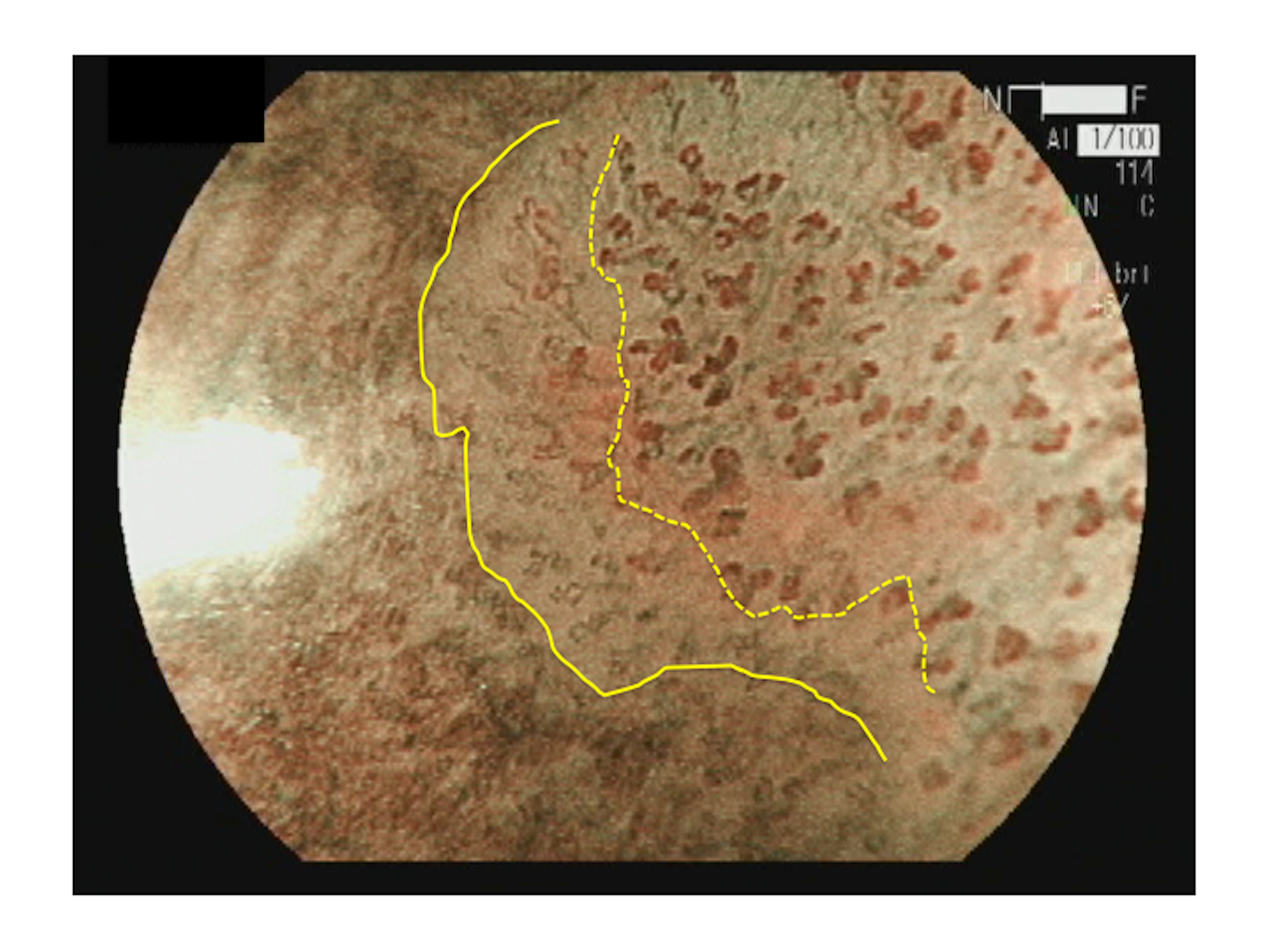

Supplement: S4 Fig — (line a: vascular line, line b: border of the iodine-unstained area) (TIFF) [file pone.0126533.s004.tiff]

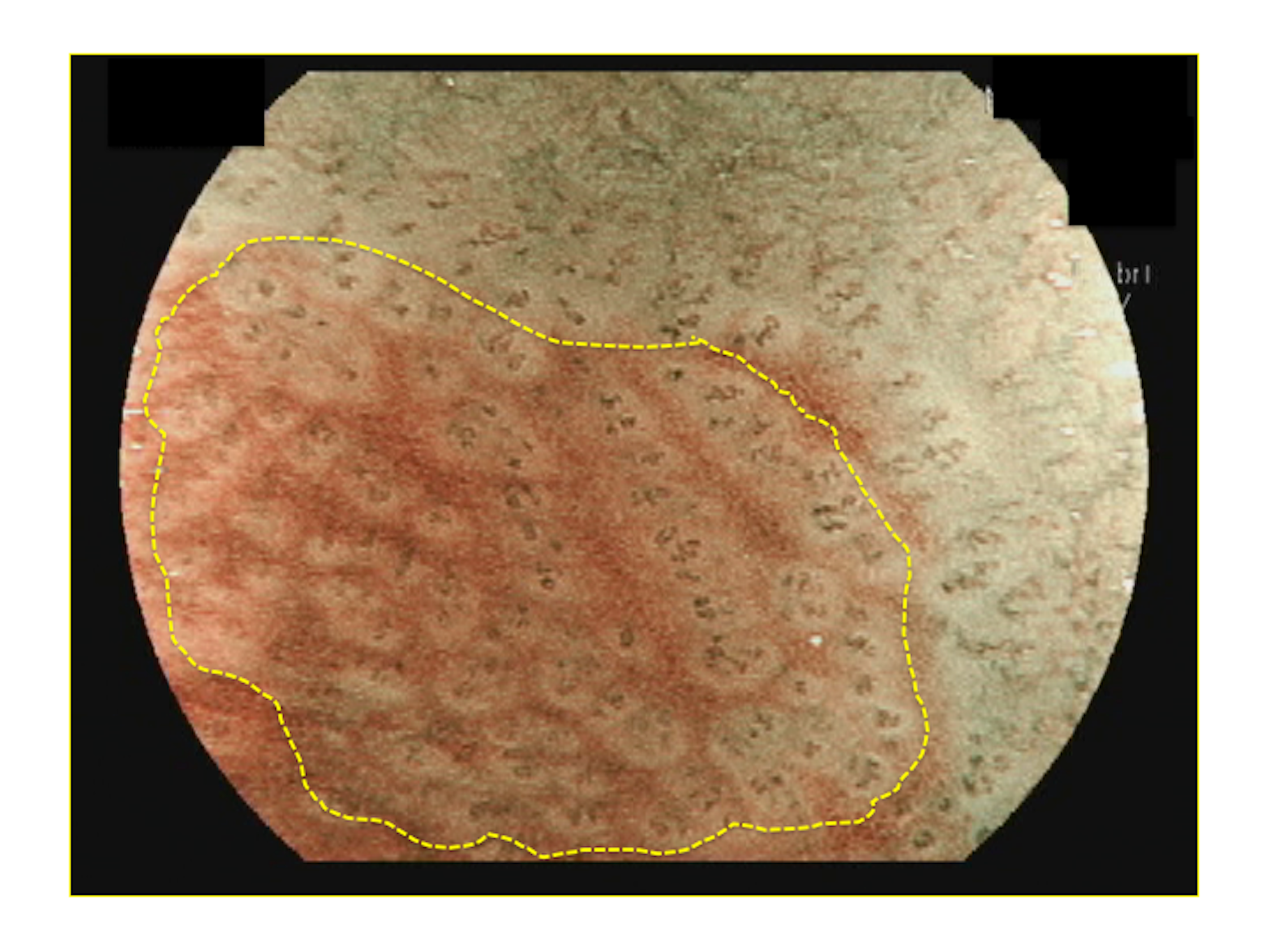

Supplement: S5 Fig — (TIFF) [file pone.0126533.s005.tiff]

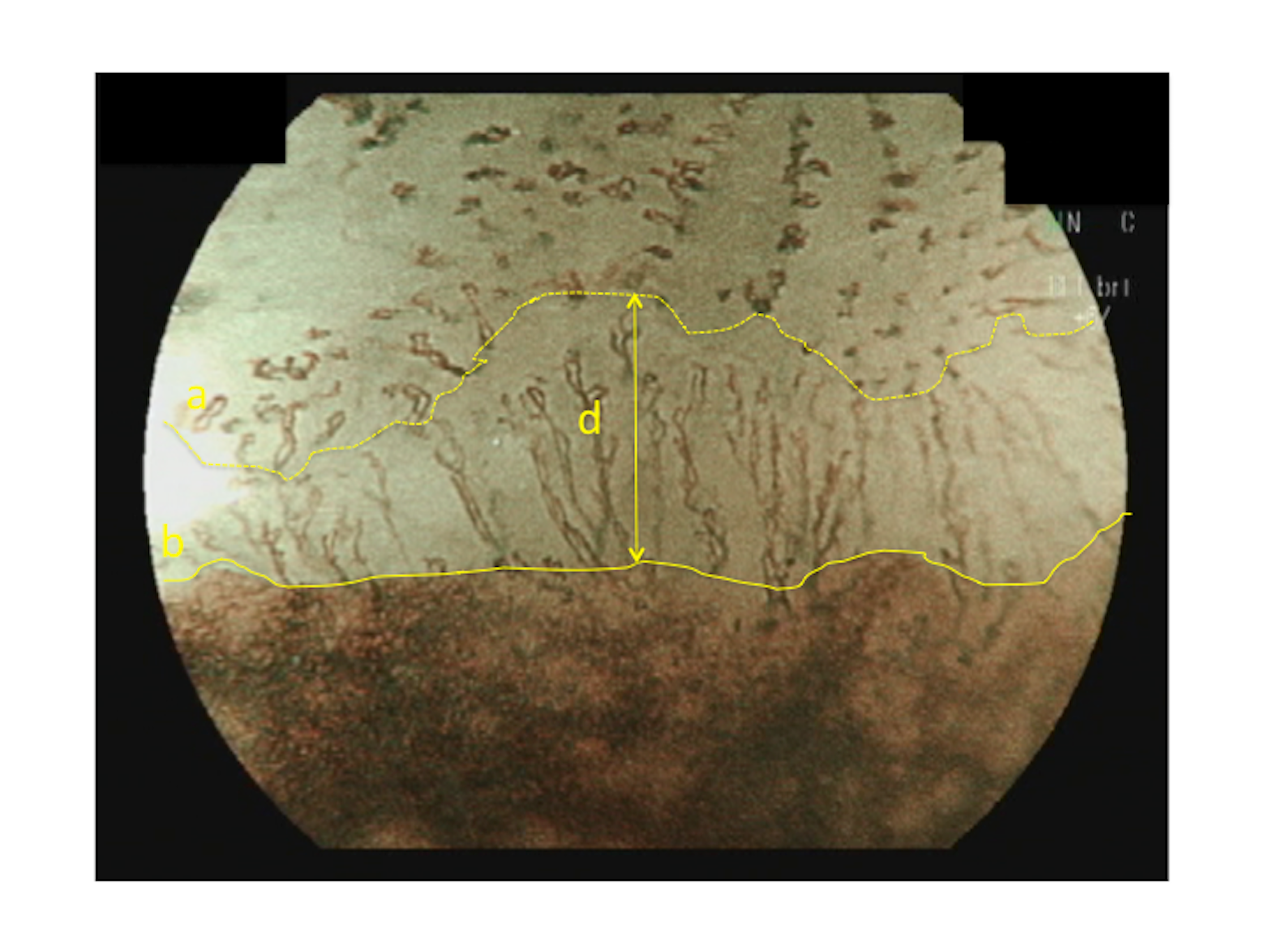

Supplement: S6 Fig — In type B, median d was 0.89 mm (range: 0.38–1.96 mm). (TIFF) [file pone.0126533.s006.tiff]

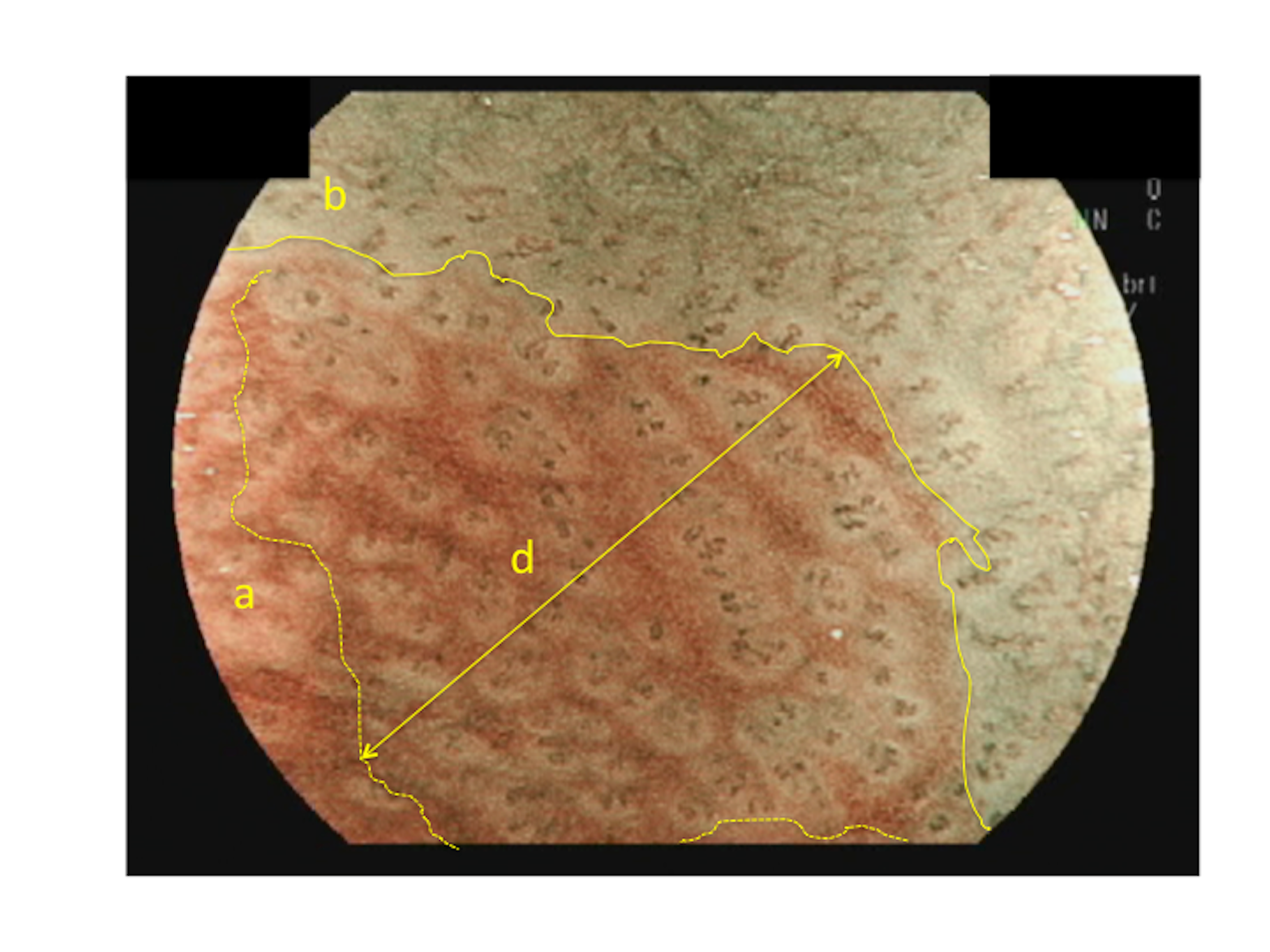

Supplement: S7 Fig — (TIFF) [file pone.0126533.s007.tiff]

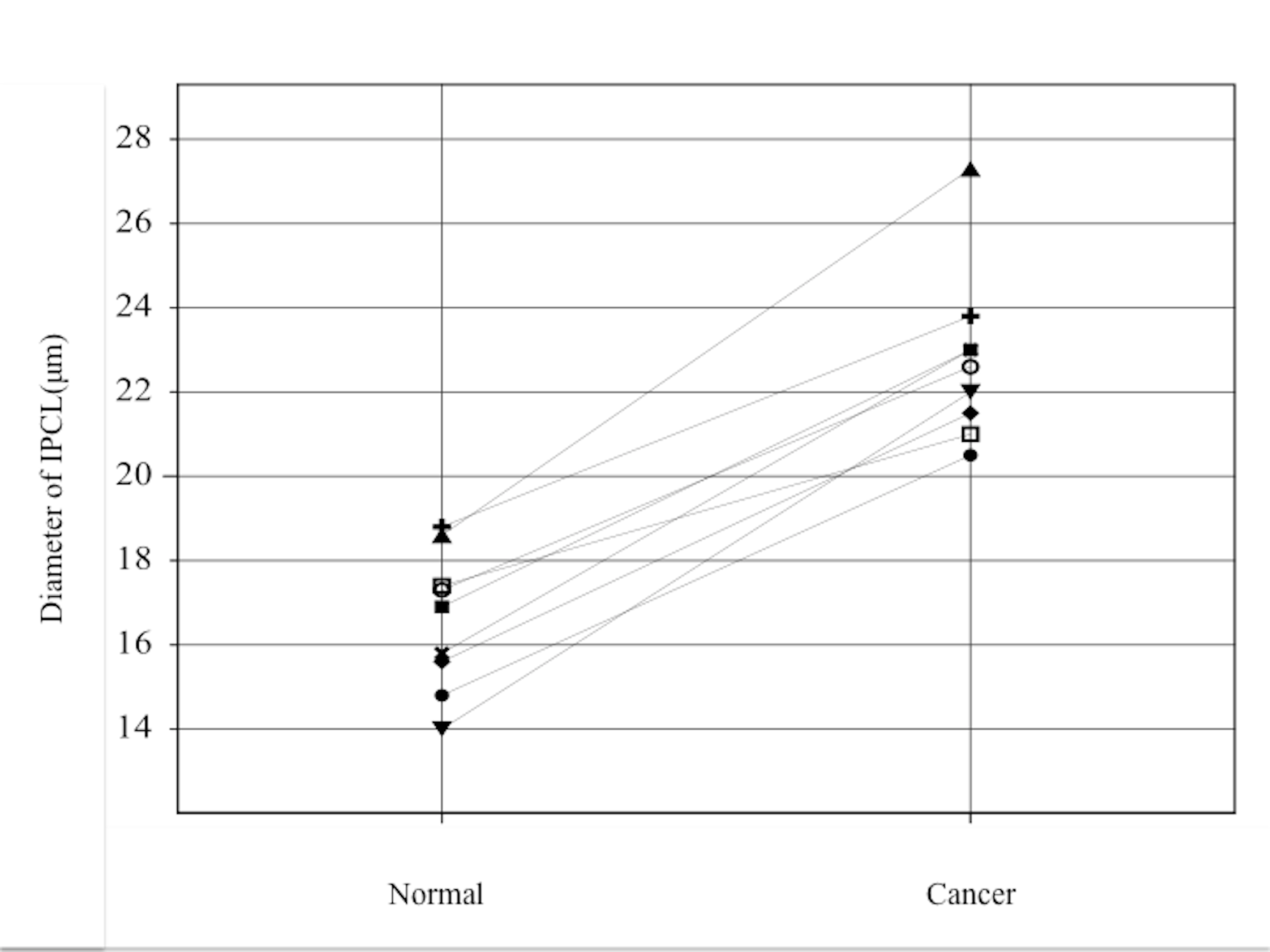

Supplement: S8 Fig — Normal IPCL: 16.2±3.7 μm (mean±SD). Cancerous IPCL: 21.9±4.4 μm (mean±SD) P<0.001, IPCL: Intra-papillary capillary loop. (TIFF) [file pone.0126533.s008.tiff]

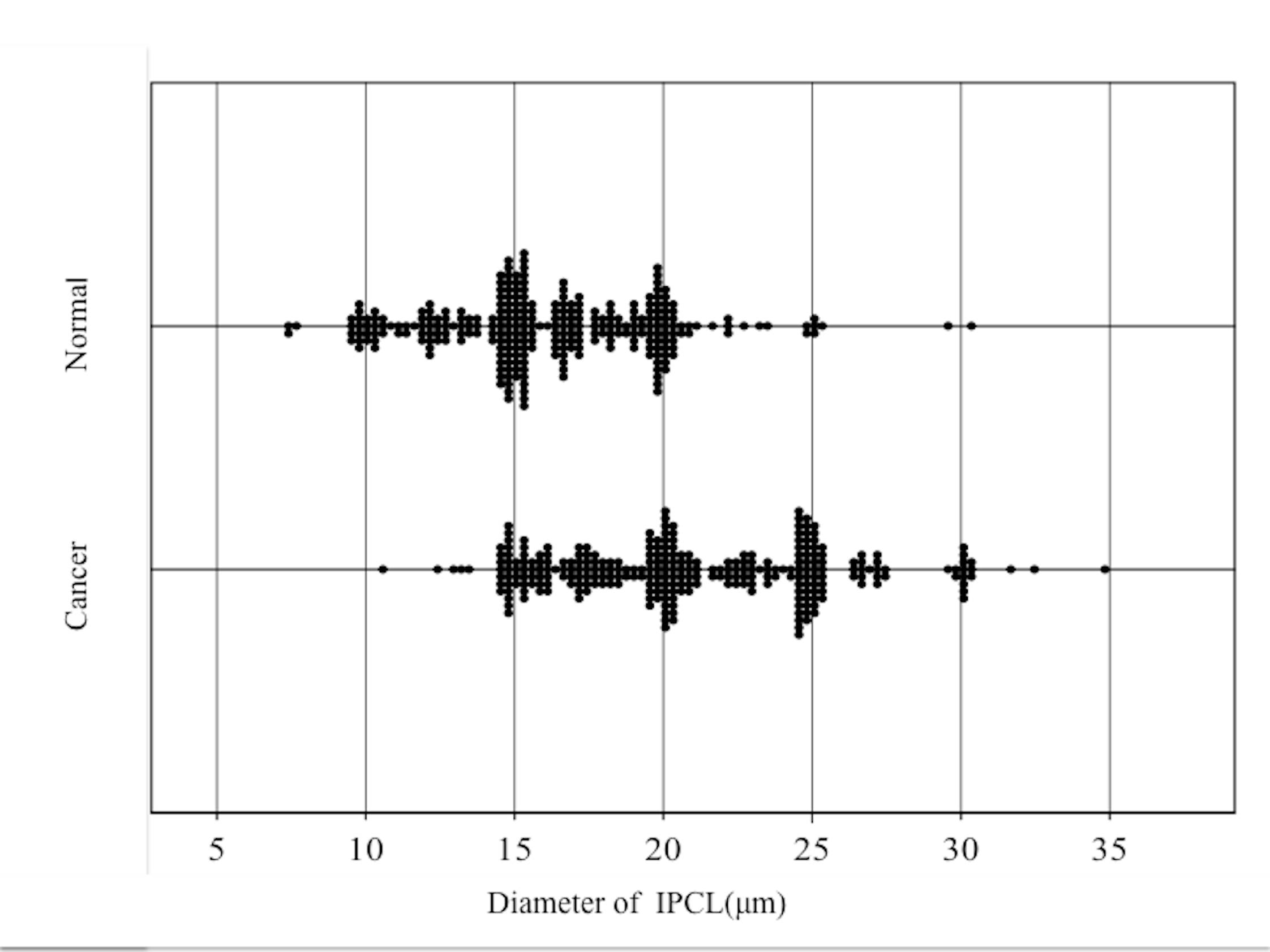

Supplement: S9 Fig — Normal IPCL: 16.0±4.0 μm (mean±SD), Cancerous IPCL: 22.2±4.2 μm (mean±SD) P<0.001, IPCL: Intra-papillary capillary loop. (TIFF) [file pone.0126533.s009.tiff]

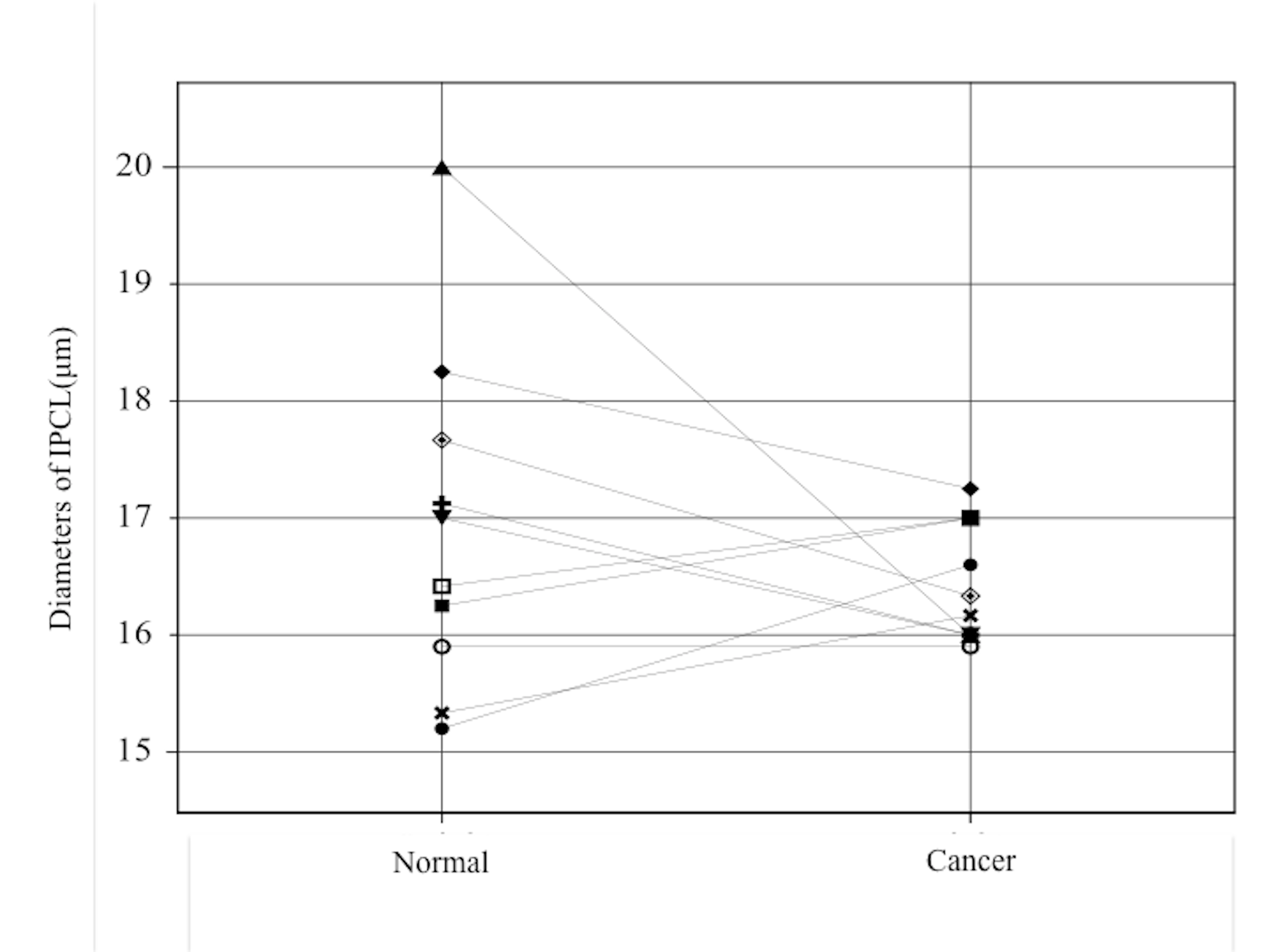

Supplement: S10 Fig — Normal IPCL: 16.5±1.7 μm (mean±SD) Cancerous IPCL: 16.5±1.4 μm (mean±SD) P = 0.35, IPCL: Intra-papillary capillary loop. (TIFF) [file pone.0126533.s010.tiff]

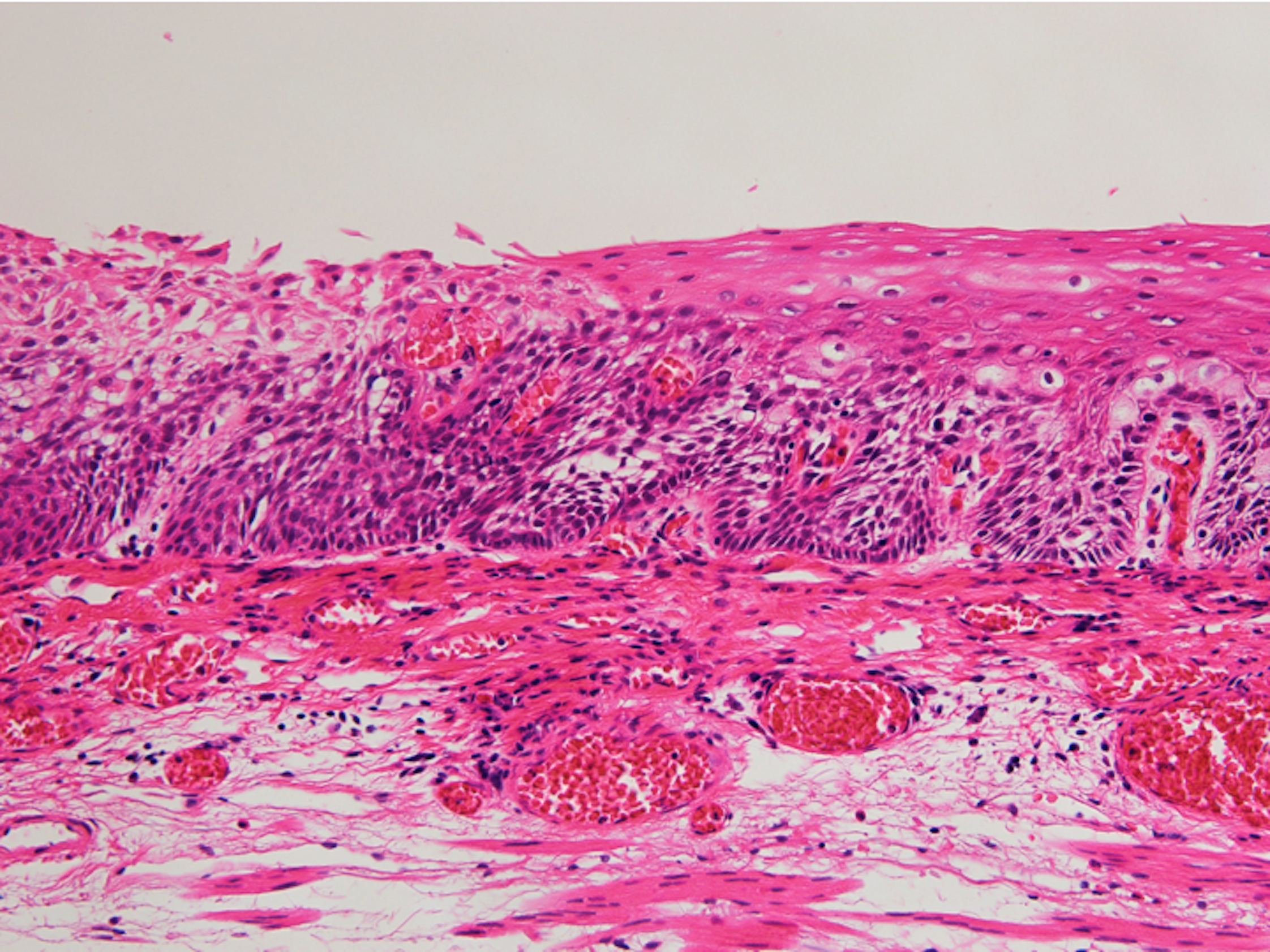

Supplement: S11 Fig — (TIFF) [file pone.0126533.s011.tiff]
